# Nanotechnology and *in Situ* Remediation: A Review of the Benefits and Potential Risks

**DOI:** 10.1289/ehp.0900793

**Published:** 2009-06-23

**Authors:** Barbara Karn, Todd Kuiken, Martha Otto

**Affiliations:** 1 U.S. Environmental Protection Agency, Washington, DC, USA; 2 Woodrow Wilson International Center for Scholars, Project on Emerging Nanotechnologies, Washington, DC, USA

**Keywords:** environmental implications, environmental technology, hazardous wastes, nano-remediation, nanotechnology, pollutants, remediation, toxicity, waste sites, zerovalent iron

## Abstract

**Objective:**

Although industrial sectors involving semiconductors; memory and storage technologies; display, optical, and photonic technologies; energy; biotechnology; and health care produce the most products that contain nanomaterials, nanotechnology is also used as an environmental technology to protect the environment through pollution prevention, treatment, and cleanup. In this review, we focus on environmental cleanup and provide a background and overview of current practice; research findings; societal issues; potential environment, health, and safety implications; and future directions for nanoremediation. We do not present an exhaustive review of chemistry/engineering methods of the technology but rather an introduction and summary of the applications of nanotechnology in remediation. We also discuss nanoscale zerovalent iron in detail.

**Data sources:**

We searched the Web of Science for research studies and accessed recent publicly available reports from the U.S. Environmental Protection Agency and other agencies and organizations that addressed the applications and implications associated with nanoremediation techniques. We also conducted personal interviews with practitioners about specific site remediations.

**Data synthesis:**

We aggregated information from 45 sites, a representative portion of the total projects under way, to show nanomaterials used, types of pollutants addressed, and organizations responsible for each site.

**Conclusions:**

Nanoremediation has the potential not only to reduce the overall costs of cleaning up large-scale contaminated sites but also to reduce cleanup time, eliminate the need for treatment and disposal of contaminated soil, and reduce some contaminant concentrations to near zero—all *in situ*. Proper evaluation of nanoremediation, particularly full-scale ecosystem-wide studies, needs to be conducted to prevent any potential adverse environmental impacts.

Nearly $1 billion for remediation projects has been allocated to the U.S. Environmental Protection Agency (EPA) in the American Recovery and Reinvestment Act (2009). Emerging technologies, such as nano-technology, could be applied in this cleanup effort to reduce costs and improve the overall effectiveness of environmental remediation methods.

“Nanotechnology is the understanding and control of matter at dimensions between approximately 1 and 100 nanometers, where unique phenomena enable novel applications” [National Nanotechnology Initiative (NNI) 2008]. Encompassing nanoscale science, engineering, and technology, nanotechnology involves imaging, measuring, modeling, and manipulating matter at this length scale. Although industrial sectors involving semiconductors; memory and storage technologies; display, optical, and photonic technologies; energy; biotechnology; and health care produce the most products containing nano-materials, there are increasing efforts to use nanotechnology as an environmental technology to protect the environment through pollution prevention, treatment, and cleanup of long-term problems such as hazardous waste sites. The technology could be a beneficial replacement of current practices for site remediation. However, potential risks are poorly understood and might lead to unintended consequences. In this review, we present a background and overview of current practice, research findings related to nanotechnology, issues surrounding the use of nanotechnology for environmental remediation, and future directions.

## Hazardous Waste Site Remediation

### Background

The U.S. Congress passed the Comprehensive Environmental Response, Compensation, and Liability Act in 1980 (CERCLA 1980). Under CERCLA, the U.S. EPA created the Superfund Program to protect human health and the environment from the risks posed by hazardous waste sites. Hundreds of thousands of sites in the United States have been identified with varied degrees of contamination. The U.S. EPA and its partners (other federal agencies and state environmental programs) continue to identify new sites every year, in addition to cleaning up sites. Under the Superfund Program, the most serious uncontrolled or abandoned hazardous waste sites have been added to the National Priorities List (NPL) for further investigation and possible remedial action. As of February 2009, the NPL contained 1,255 sites (U.S. EPA 2009).

### Scope of the cleanup market

In fiscal year 2007 alone, the Superfund Program spent $380 million for construction and post-construction activities for site remediation projects (U.S. EPA 2008a). The Superfund Program, which includes the NPL, is just one of many cleanup programs, for example, the Brownfields Program [under the Small Business Liability Relief and Brownfields Revitalization Act (2002)], corrective action (CA) programs under Subtitle C of the Resource, Conservation, and Recovery Act (RCRA 2002), and the Underground Storage Tank program under Subtitle I of the RCRA.

The U.S. EPA, U.S. Department of Defense (DOD), U.S. Department of Energy (DOE), other federal agencies, state environmental agencies, corporations, and private parties all may conduct site cleanups. The same suite of remediation technologies is generally used at sites, regardless of the regulatory program under which they fall (U.S. EPA 2004). As part of the American Recovery and Reinvestment Act (2009), the U.S. EPA was allocated $600 million for the Superfund Remedial Program, $200 million for the Leaking Underground Storage Tank Trust Fund Program, and $100 million for the Brownfields Program.

To date, only a small fraction of site remediation has been conducted by the U.S. EPA. Most cleanup is funded by public and private property owners who are potentially responsible for the contamination (U.S. EPA 2004). The U.S. EPA (2004) estimated that it will take 30–35 years and cost up to $250 billion to clean up the nation’s hazardous waste sites. The U.S. EPA (2004) anticipates that these high costs will provide an incentive to develop and implement cleanup approaches and technologies that will result in “better, cheaper, and faster site cleanups.” Developing cost-effective, *in situ* groundwater treatment technologies could save billions of dollars in cleanup costs.

**Figure 1:**
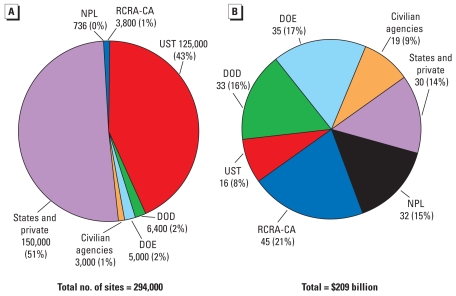
Estimated number (%) of U.S. hazardous waste sites (*A*) and estimated cleanup costs [billions US$ (percent of total)] for 2004–2033 (*B*). UST, underground storage tanks. Adapted from U.S. EPA (2004).

Figure 1A shows U.S. EPA estimates of the total number of hazardous waste sites in the United States (U.S. EPA 2004). State and private property owners make up the largest percentage (51%), followed by sites with underground storage tanks (43%). Together, they account for nearly 94% of all hazardous waste sites in the United States. Most of these sites have similar contaminants, such as solvents and other organics, metals, and petroleum products. Although DOD and DOE sites constitute < 4% of the total number of sites, they tend to be larger and more complex. Figure 1B shows estimates for the total cleanup costs associated with these sites. Although state and private-party sites make up 51% of the total hazardous waste sites, they represent only 14% of the total costs. Most of the estimated costs to remediate U.S. hazardous wastes sites are borne by RCRA-CA (21%), DOE (17%), and DOD (16%) sites. Together, these add up to $89 billion, or 54% of the total market.

More than 80% of NPL sites have contaminated groundwater. This is particularly important considering that more than half of the U.S. population relies on groundwater for drinking. Once groundwater is polluted, its remediation is often protracted, costly, and sometimes infeasible.

### Pump and treat

Early treatment remedies for groundwater contamination were primarily pump-and-treat operations. This method involves extracting contaminated groundwater via wells or trenches and treating the groundwater above ground (*ex situ*) using processes such as air stripping, carbon adsorption, biological reactors, or chemical precipitation (U.S. EPA 2001). Many of these processes produce highly contaminated wastes that then have to be disposed.

The U.S. EPA (2001) studied the average operating costs of pump-and-treat systems at 32 Superfund-financed sites and found the annual cost to be approximately $767,000/site. The average pump-and-treat system operated for 5 years, treating an average 118 million gallons of water per site for an average cost of $9.4 million to clean up a single site (U.S. EPA 2001). Many of these sites have ongoing monitoring, which continues to incur annual operating costs. Sites contaminated with nonaqueous-phase liquids (NAPLs) tend to operate for longer periods of time, incurring even higher average costs (U.S. EPA 2004).

Pump-and-treat projects represent the largest number of treatments at Superfund sites, 38% (725 of 1,915). Of the 1,915 treatment remedies tracked by the U.S. EPA, 36% (687 projects) have been completed or shut down. However, only 11% of these 687 projects are pump-and-treat projects (U.S. EPA 2008a).

### *In situ* remediation

A common type of *in situ*, or below-ground, remediation method currently used to clean up contaminated groundwater is the permeable reactive barrier (PRB). PRBs are treatment zones composed of materials that degrade or immobilize contaminants as the groundwater passes through the barrier. They can be installed as permanent, semipermanent, or replaceable barriers within the flow path of a contaminant plume. The material chosen for the barrier is based on the contaminant(s) of concern (U.S. EPA 2001). One drawback of PRBs is that they can only remediate contaminant plumes that pass through them; they do not address dense NAPLs (DNAPLs) or contaminated ground-water that is beyond the barrier.

Other *in situ* treatment technologies include thermal treatment (steam-enhanced extraction, electrical resistive heating, or thermal conductive heating), chemical oxidation, surfactant cosolvent flushing, and bioremediation.

Because of the high cost and lengthy operating periods for pump-and-treat remedies, use of *in situ* groundwater treatment technologies is increasing. Remedies selected for NPL sites are documented in records of decision (RODs). A ROD provides the justification for the remedial action (treatment) chosen at a Superfund site. The percentage of RODs that selected *in situ* groundwater treatment went from zero in fiscal years 1982–1986 to 31% in fiscal year 2005. RODs that select pump and treat (*ex situ*) alone have decreased from about 80% before fiscal year 1992 to an average of 20% during fiscal years 2001–2005 (U.S. EPA 2008a). *Ex situ* remediation techniques could be phased out over the coming decade.

## Nanoremediation

Nanoremediation methods entail the application of reactive nanomaterials for transformation and detoxification of pollutants. These nanomaterials have properties that enable both chemical reduction and catalysis to mitigate the pollutants of concern. For nanoremediation *in situ*, no groundwater is pumped out for above-ground treatment, and no soil is transported to other places for treatment and disposal (Otto et al. 2008).

Nanomaterials have highly desired properties for *in situ* applications. Because of their minute size and innovative surface coatings, nanoparticles may be able to pervade very small spaces in the subsurface and remain suspended in groundwater, allowing the particles to travel farther than larger, macro-sized particles and achieve wider distribution. However, in practice, current nanomaterials used for remediation do not move very far from their injection point (Tratnyek and Johnson 2006).

Many different nanoscale materials have been explored for remediation, such as nanoscale zeolites, metal oxides, carbon nanotubes and fibers, enzymes, various noble metals [mainly as bimetallic nanoparticles (BNPs)], and titanium dioxide. Of these, nanoscale zerovalent iron (nZVI) is currently the most widely used. The different nanomaterials, along with the pollutants they could potentially remediate, are listed in Supplemental Material, Table 1, available online (doi:10.1289/ ehp.0900793.S1 via http://dx.doi.org/). [For a comprehensive overview of the chemistry and engineering of various nanotechnology applications addressed in Supplemental Material, Table 1, and used for remediation, see Theron et al. (2008) and Zhang (2003).]

### nZVI

nZVI particles range from 10 to 100 nm in diameter, although some vendors sell micrometer-scale iron powders as “nanoparticles.” Typically, a noble metal (e.g., palladium, silver, copper) can be added as a catalyst. The second metal creates a catalytic synergy between itself and Fe and also aids in the nanoparticles’ distribution and mobility once injected into the ground (Saleh et al. 2007; Tratnyek and Johnson 2006; U.S. EPA 2008b). These BNPs may contain more than two different metals. The second metal is usually less reactive and is believed to promote Fe oxidation or electron transfer (U.S. EPA 2008b). Some noble metals, particularly palladium, catalyze dechlorination and hydrogenation and can make the remediation more efficient (U.S. EPA 2008b; Zhang and Elliott 2006). The underlying chemistry of the reaction of Fe with environmental pollutants (particularly chlorinated solvents) has been extensively studied and applied in micrometer-scale ZVI PRBs (Matheson and Tratnyek 1994). There are two main degradation pathways for chlorinated solvents: beta elimination and reductive chlorination. Beta elimination occurs most frequently when the contaminant comes into direct contact with the Fe particle. The following example shows the pathway of trichloroethene (TCE):


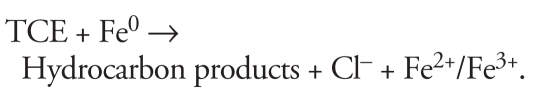


Under reducing conditions fostered by nZVI in groundwater, the following reaction takes place:


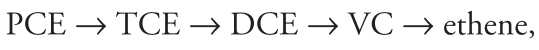


where PCE is perchloroethylene, DCE is dichloroethylene, and VC is vinyl chloride (Tratnyek 2003, U.S. EPA 2008b).

In the 1990s, Fe at the nanoscale was synthesized from Fe(II) and Fe(III) to produce particles ranging from 10 to 100 nm, initially using borohydride as the reductant, and examined in laboratory studies. Zhang (2003) tested nZVI for the transformation of a large number of pollutants, most notably halogenated organic compounds commonly detected in contaminated soil and groundwater. The author reported that nanoscale Fe particles are very effective for the transformation and detoxification of a variety of common environmental pollutants, including chlorinated organic solvents, organochlorine pesticides, and polychlorinated biphenyls (PCBs). According to Zhang (2003), Fe-mediated reactions should produce an increase in pH and a decrease in the solution redox potential created by the rapid consumption of oxygen, other potential oxidants, and the production of hydrogen. Although batch reactors produce pH increases of 2–3 and an oxidation–reduction potential (ORP) range of −500 to −900 mV, it is expected that the pH and ORP would be less dramatic in field applications where other mechanisms reduce the chemical changes (Zhang 2003). Previous work showing an increase of pH by 1 and an ORP in the range of −300 to −500 mV supports this assessment (Elliott and Zhang 2001; Glazier et al. 2003). Zhang (2003) also showed that modifying Fe nanoparticles could enhance the speed and efficiency of the remediation process.

The first field application was reported in 2000 (Zhang 2005). Nanoparticles have been shown to remain reactive in soil and water for up to 8 weeks and can flow with the ground-water for > 20 m. In one study, Zhang (2003) produced a 99% reduction of TCE within a few days of injection.

Because nanoscale particles are so small, Brownian movement or random motion, rather than wall effects, dominates their physical movement or transport in water. The movement of micrometer-scale particles, especially microscale metal particles, is largely controlled by gravity-induced sedimentation because of their size and high density. In the absence of significant surface electrostatic forces, nanosized particles can be easily suspended in water during the design and manufacturing stages, thus providing a versatile remediation tool that allows direct injection as a liquid into the subsurface where contaminants are present. Coating the Fe particles to improve mobility and catalytic reaction rates is important. Some of the particles flow with the groundwater and remain in suspension for various amounts of time, whereas others are filtered out and bind to soil particles, providing an *in situ* treatment zone that could hold back emanating plumes (Henn and Waddill 2006).

The high reactivity of nZVI particles is in part a direct result of their high specific surface area. For example, nZVI produced by the borohydride method has surface areas in the range of 20–40 m^2^/g, which can yield 10–1,000 times greater reactivity compared with granular Fe, which has a surface area < 1 m^2^/g (Wang and Zhang 1997). nZVI’s small particle size also allows more of the material to penetrate into soil pores, and it can be more easily injected into shallow and deep aquifers, a property that is particularly beneficial when contamination lies underneath a building.

Initially, Fe nanoparticles have a core of ZVI and an outer shell of Fe oxides, which suggest the following redox reactions:


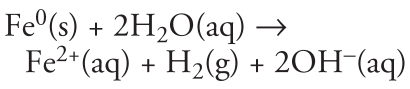



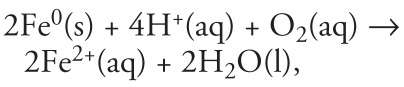


where s is solid, aq is aqueous, g is gas, and l is liquid (Matheson and Tratnyek 1994).

Although Fe nanoparticles have been shown to have a strong tendency to form microscale aggregates, possibly because of their weak surface charges, coatings can be applied to change the surface properties. These different forms of Fe could be useful for the separation and transformation of a variety of contaminants, such as chlorinated organic solvents, organochlorine pesticides, PCBs, organic dyes, various inorganic compounds, and the metals As(III) (trivalent arsenic), Pb(II) (bivalent lead), copper [Cu(II) (bivalent copper)], Ni(II) (bivalent nickel), and Cr(VI) (hexavalent chromium) (Sun et al. 2006).

Nanoremediation, particularly use of nZVI, has site-specific requirements that must be met in order for it to be effective. Adequate site characterization is essential, including information about site location, geologic conditions, and the concentration and types of contaminants. Geologic, hydrogeologic, and subsurface conditions include composition of the soil matrix, porosity, hydraulic conductivity, groundwater gradient and flow velocity, depth to water table, and geochemical properties (pH, ionic strength, dissolved oxygen, ORP, and concentrations of nitrate, nitrite, and sulfate). All of these variables need to be evaluated before nanoparticles are injected to determine whether the particles can infiltrate the remediation source zone, and whether the conditions are favorable for reductive transformation of contaminants. The sorption or attachment of nanoparticles to soil and aquifer materials depends on the surface chemistry (i.e., electrical charge) of soil and nanoparticles, groundwater chemistry (e.g., ionic strength, pH, and presence of natural organic matter), and hydrodynamic conditions (pore size, porosity, flow velocity, and degree of mixing or turbulence). The reactions between the contaminants and the nZVI depend on contact or probability of contact between the pollutant and nanoparticles (U.S. EPA 2007, 2008b).

In field tests, Henn and Waddill (2006) found that, with the use of nZVI, decreases in parent pollutant compound concentrations (TCE and trichloroethane) were accompanied by increases and subsequent decreases in daughter product concentrations (*cis*-1,2-DCE, 1,1-dichloroacetic acid, 1,1-DCE, and vinyl chloride). Long-term observations indicated that although the degradation was continuous, it was at a much slower rate for the daughter products. Their study concluded that there was overall reduction in contaminants, a reduced plume size, and reduction in the contaminant mass flux emanating from the source. The nanoscale Fe created conditions for abiotic degradation for about 6–9 months, followed by biological degradation as the primary degradation process. Both processes had significant impacts on the degradation of contaminants (Henn and Waddill 2006). Cao et al. (2005) found that nZVI particles in an aqueous solution reduced perchlorate to chloride almost completely without producing intermediate degradation products.

Fe oxide nanoparticles have been shown to bind As irreversibly up to 10 times more effectively than micrometer-sized particles. Based on their super-paramagnetic properties, the Fe particles and bound As can be separated from the water with a magnetic field. Laboratory tests have shown 99% removal of As using 12-nm-diameter Fe oxide nanoparticles (Rickerby and Morrison 2007). Kanel et al. (2006) concluded that nZVI can reduce As(V) to As(III) in a short period of time at neutral pH. They also found that a high amount of nZVI was needed to completely remove As(V), possibly because of the presence of dissolved organic carbon, sulfate, and phosphate.

The hydrophilic properties of nZVI enable the remediation of aqueous-phase contaminants, including DNAPLs. Because the addition of nZVI in the source zone reduces contaminants, it increases the concentration gradient between the aqueous phase and DNAPLs, thereby increasing the mass transfer of contaminants from DNAPLs to the dissolved aqueous phase, where they are then treated (Watlington 2005). To address DNAPLs directly, emulsified ZVI (eZVI) has been used. When the emulsion droplets come into contact with dissolved TCE, the contaminant diffuses into the interior of the emulsion droplet, where it comes into contact with the ZVI and is degraded. A concentration gradient is established by migration of the TCE molecules into the interior aqueous phase of the emulsion droplet and by migration of the by-products out of the droplet and into the surrounding water phase, further driving the degradation reactions (O’Hara et al. 2006).

In other field test research conducted between 2003 and 2005 in North America and Europe, nZVI was effective in treating various compounds in groundwater, including chlorinated solvents and Cr(VI) (Macé et al. 2006). These field tests showed that the concentrations of chlorinated solvents decreased dramatically during the first few hours and days after injection and remained low in conjunction with the mass balance of nZVI versus the mass of chlorinated hydrocarbons in the remediation area. When present, sulfates and nitrates also decreased in parallel with the chlorinated solvents, but diminished the effect of nZVI on other solvents.

Macé et al. (2006) found that nZVI moved with groundwater away from the injection site. Based on this, they hypothesized that nZVI could treat larger areas of the affected aquifers. They found dramatic but short-lived reductions of volatile organic compounds (VOCs) in fractured bedrock and a slower, steadier decrease of VOCs in primary porosity aquifers. The same study suggested that the degradation of VOCs and travel velocity are indirectly proportional to the hydraulic conductivity. BNPs reacted more quickly and were spent more rapidly than nZVI, whereas nZVI reacted more slowly but had a longer effect. Macé et al. (2006) noted minor but inconclusive changes to the microbial community due to the addition of nanoparticles. These changes could affect parallel bioremediation.

In an extensive study, the Navy conducted field tests using nZVI to remediate two of its contaminated sites (Naval Air Engineering Station, Lakehurst, NJ, and Naval Air Station, Jacksonville, FL) and using micrometer-sized ZVI powder at a third site, Hunters Point Shipyard, Hunters Point, California (Gavaskar et al. 2005). In the Jacksonville study, TCE concentrations in a well approximately 20 ft from the source zone were reduced up to 99%, suggesting that some of the nZVI migrated outside of the treatment zone through preferential pathways. Long-term monitoring of the treatment zone was recommended to demonstrate that the decline in parent compounds (e.g., TCE) and by-products (e.g., *cis*-1,2-DCE) persists after the ZVI is depleted, which will permit determination of how much, if any, DNAPL mass truly remains in the treatment zone (Gavaskar et al. 2005).

In addition to groundwater remediation, nanotechnology holds promise in reducing the presence of NAPLs. Recently, a material using nanosized oxides (mostly calcium) was used *in situ* to clean up heating oil spills from underground oil tanks. Preliminary results from this redox-based technology suggest faster, cheaper methods and, ultimately, lower overall contaminant levels compared with previous remediation methods. Most of these sites have been in New Jersey, with cleanup conducted in consultation with the New Jersey Department of Environmental Protection (see Continental Remediation LLC 2009).

### The state of the practice

**Figure 2:**
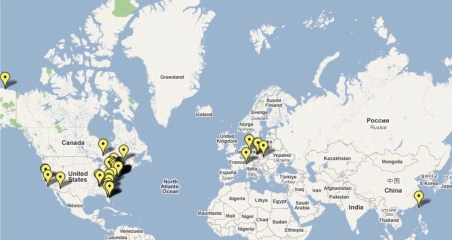
Map of remediation sites listed in Supplemental Material, Table 2 (doi:10.1289/ehp.0900793.S1) (Project on Emerging Nanotechnologies 2009).

The number of actual applications of nZVI is increasing rapidly. Only a fraction of the projects has been reported, and new projects show up regularly. Figure 2 and Supplemental Material, Table 2 (doi:10.1289/ehp.0900793.S1) describe 44 sites where nanoremediation methods have been tested for site remediation. These sites are in seven countries (including the United States) and in 12 U.S. states. All of the sites have some form of chlorinated compounds of concern, such as PCE, TCE, or PCBs. Other pollutants include Cr(VI) and nitrate. The sites include oil fields, manufacturing sites, military installations, private properties, and residences.

**Figure 3:**
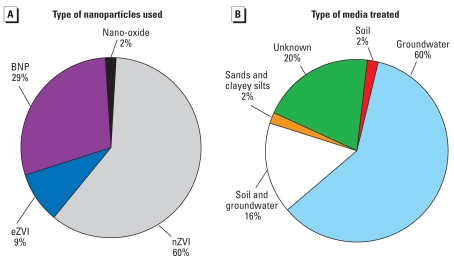
Type of nanoparticles used (*A*) and type of media treated (*B*) at sites listed in Supplemental Material, Table 2 (doi:10.1289/ehp.0900793.S1).

Figure 3A shows the types of manufactured nanoparticles used for remediation at the sites. More than two-thirds of the sites are treated with a form of ZVI, and most of the BNPs contain Fe. Figure 3B shows the types of media treated at these sites. More than three-fourths of the sites treated contain contaminated groundwater.

Supplemental Material, Table 2 (doi:10. 1289/ehp.0900793.S1) lists details of the 44 sites treated with nanomaterials and the results of the treatment. Because data from most of these sites were not collected as part of a research project, the information is general and, in most cases, incomplete. For example, site 2 is a BP Global site in Alaska contaminated with trichloroethane; when BNPs were used, practitioners saw reductions of 60% and 90% for shallow test and deep test concentrations, respectively. At site 11, in the Czech Republic, nZVI was used to reduce chlorinated solvents. Levels were reduced to an order of magnitude lower than original concentrations and were maintained for 6 months. Supplemental Material, Table 2, provides an overview of the current state of the practice using nanomaterials, mainly ZVI, for site remediation.

Because many of the remediation projects using nanoparticles are just beginning or are ongoing, cost and performance data are limited. However, as the technology is applied at an increasing number of sites with varying geologies, more data will become available on performance, cost, and environmental aspects, thereby providing site managers and other stakeholders with additional information to determine whether the technology might be applicable to their specific sites.

PARS Environmental Inc. (2004) conducted a case-study cost comparison of a manufacturing site in New Jersey where the primary contaminants of concern were TCE and PCE. They estimated that using the pump-and-treat method would cost approximately $4,160,000 and PRB approximately $2,200,000. nZVI would cost approximately $450,000, representing a cost savings of 80–90% over the pump-and-treat method.

Table 1 indicates the relative magnitude of the media and contaminant group at four types of remediation sites. Using Table 1 and Figure 1B, the cost savings to remediate groundwater can be estimated for NPL, RCRA, DOD, and DOE sites. Using nano-remediation, potential savings of $87 billion to $98 billion can be realized to clean up the nation’s hazardous waste sites over the next 30 years. Although this estimate is based on publicly available data and assumes use of nZVI or a variation of nanoremediation for all sites with contaminated groundwater, it is a reasonable estimate of the magnitude of cost savings achievable using this technology. Increased manufacturing capacity to supply the amount of nanomaterials needed could lead to lower costs from economies of scale. It should be noted, however, that not all sites have conditions suitable for nanoscale remediation methods.

In addition to the potential cost savings associated with using nanotechnology for site remediation, the amount of time required to clean up a site could be greatly reduced. The average pump-and-treat system operates for about 18 years (U.S. EPA 2001). In a study using nZVI, Zhang (2003) observed a 99% reduction in TCE levels within days of injection. This shortened time interval not only reduces operating costs but also reduces the time that workers are exposed to a contaminated site during cleanup. Environmental disturbances that can affect the local ecosystem’s flora, fauna, and microorganisms are reduced because nZVI is injected using small wells instead of excavating soils or removing groundwater using pump-and-treat methods; the time of site disturbance is also shorter.

## Potential Implications

### Fate and transport

When released into the environment, manufactured nanoparticles aggregate to some degree and behave like natural nanomaterials. However, to be effective, nZVI needs to form stable dispersions in water so it can be delivered to water-saturated porous material in the contaminated area. Yet, its rapid aggregation limits its mobility (Phenrat et al. 2007). The rapid aggregation of the nanoscale Fe particles supports the need for polymer or other coatings to modify the nZVI surface in order to improve mobility (Phenrat et al. 2007).

Depending on the composition of ground-water and the hydrologic conditions, certain nanoscale colloids have the ability to travel unexpectedly large distances in the environment (Kersting et al. 1999; Novikov et al. 2006; Vilks et al. 1997). They could form stable nanoclusters in groundwater that are likely to be highly mobile, carrying with them surface-sorbed contaminants. These natural particles can carry materials between redox zones and facilitate or inhibit contaminant transport (Waite et al. 1999).

The mobility of natural or synthetic nano-particles in the natural environment will strongly depend on whether the nanoparticles remain completely dispersed, aggregate and settle, or form mobile nanoclusters. Gilbert et al. (2007) suggested that many manufactured metal oxide and other inorganic nano-particles will exhibit cluster-forming behavior similar to that of natural nanoparticles. Despite numerous observations that nanoscale minerals represent an important fraction of the environmental colloids, the fundamental aggregation and transport properties of nanoparticles have not been extensively studied.

In addition to self-aggregation, nano-particles could associate with suspended solids or sediment, where they could bioaccumulate and enter the food chain or drinking water sources. These fate processes depend on both the characteristics of the particle and the characteristics of the environmental system (Boxall et al. 2007).

The use of nanoparticles in environmental remediation will inevitably lead to the release of nanoparticles into the environment and subsequent ecosystems. To understand and quantify the potential risks, the mobility, bioavailability, toxicity, and persistence of manufactured nanoparticles need to be studied (Nowack 2008). To pose a risk, nano-particles must be hazardous and have a route for exposure. Although aggregated and/or absorbed nanoparticles are usually less mobile, they still have the potential to be taken up by filter feeders and other sediment-dwelling organisms. The U.S. EPA has raised the possibility of biomagnification of nanoparticles; however, no data currently exist proving or disproving this hypothesis (Biswas and Wu 2005; U.S. EPA 2007). To be able to quantify the stability of nanoparticles in the environment, the stability of their suspensions and their tendency to aggregate and interact with other particles must first be determined (Mackay et al. 2006).

### Potential toxicity

Naturally occurring nanoscale Fe oxide particles with metals (such as copper) bound to their surface have been found many kilometers downstream from mining sites, indicating the ability of these colloidal nanoparticles to move and transport sorbed contaminants (Hochella et al. 2005). These binding properties and processes may show size-dependent reactivity on crystalline Fe oxide nanoparticles, and each process might occur with different thermochemical and kinetic relationships as a function of size (Madden et al. 2006). Thus, whereas the nanoparticles themselves may not possess toxic properties, the pollutants they could carry with them may. Fe nanomaterials may bind with and carry copper, which has a toxicity threshold for algae, flowering plants, fungi, and phytoplankton that is surpassed only by mercury and sometimes silver (Sposito 1989).

Handy et al. (2008) suggested that despite the environment containing many natural particles at the nanoscale, manufactured nano-particles may act differently. These materials are designed to have specific surface properties and chemistries that are not likely to be found in natural particles. The properties of manufactured nanoparticles enhance novel physico-chemical and possibly toxicologic properties compared with natural particles. A range of ecotoxicologic effects of various manufactured nanomaterials has been reported, including effects on microbes, plants, invertebrates, and fish (Boxall et al. 2007). Laboratory studies using fish, *Daphnia*, copepods, and other organisms (Adams et al. 2006; Fortner et al. 2005; Lovern et al. 2007; Oberdörster et al. 2006) have shown that these organisms can take up some manufactured nanoparticles.

The factors and processes affecting eco-toxicity are complex, and the impact of manufactured nanoparticles on organisms is determined by a range of properties, including dissolution potential, aggregation potential, particle surface properties, the characteristics of the exposure environment, and the biochemical, physiological, and behavioral traits of the organism being exposed (Dhawan et al. 2006). Although available data indicate that current risks of manufactured nanoparticles in the environment to environmental and human health are probably low (see Table 3 in Boxall et al. 2007), knowledge of their potential impact in the environment and on human health is still limited.

Research on ultrafine particulates (< 100 nm in one dimension) has shown that as particle size decreases, potential for pulmonary toxicity tends to increase even if the material’s larger form is inert. nZVI is typically between tens and hundreds of nanometers in size at the time of production. Under laboratory conditions, these particles tend to aggregate and produce clusters that can build up to the micrometer size. If this occurs, they will not take on the properties that apply to actual nanosized particles and will behave similarly to larger environmental colloids (Tratnyek and Johnson 2006).

Inhalation exposure to Fe^0^(s) nanoparticles could result in the release of Fe(III), followed by oxidative damage due to generation of Fe(IV) (Keenan and Sedlak 2008). *In vitro* studies examining the response of the central nervous system to low concentrations of nano-Fe and nanomagnetite showed that these nanoparticles are taken up into cells and produce an oxidative stress response (Wiesner et al. 2006). These studies indicate a potential for adverse health effects from exposure and uptake of Fe oxide nanoparticles into mammalian cells. The authors caution, however, that these tests were conducted at much higher dosages than would be encountered normally (Wiesner et al. 2006).

In some cases, Fe oxide nanoparticles (a potential end product from redox reactions of nZVI) can be internalized by cells and cause cell death. Low solubility of Fe oxide nano-particles enables them to persist in biological systems and could potentially induce long-term effects involving mutagenic influence on organisms (Auffan et al. 2006). However, there are limited data on the interactions of Fe oxide nanoparticles with cells and the effect that coatings can have on cell adhesion, internalization, and interaction.

Mineral nanoparticles are common components of natural aqueous systems. Several natural inorganic and biologically mediated processes produce mineral nanoparticles, such as metal sulfides and metal oxides (Labrenz et al. 2000; Villalobos et al. 2003). Nanoscale Fe (oxy)hydroxide phases are among the most common natural mineral nanoparticles formed by precipitation from solution after oxidation of aqueous ferrous Fe (Van der Zee et al. 2003). Although Fe is an essential element for growth in nearly all species, an abundance of free chelating Fe has been linked to DNA damage, lipid peroxidation, and oxidative protein damage *in vivo* (Valko et al. 2005).

Particle coating, surface treatments, surface excitation by ultraviolet radiation, and particle aggregation can modify the effects of particle size, suggesting that some nanoparticles could exert their toxic effects as aggregates or through the release of toxic chemicals (Nel et al. 2006). Although the aggregates are fractal-like, they may exhibit some of the properties of the discrete nanoparticles, including specific surface area and reactivity, particularly because these particles have been manufactured at the nanoscale in order to harness particular nanoscale properties.

Generally, little concern has been raised about the toxicity of nZVI because Fe oxides formed during remediation are already present in the form of rust and because the nano-Fe particles have not been found to produce radically new properties, compared with microscale-sized Fe particles (Watlington 2005). Whether the addition of catalytic coatings changes these properties or presents another hazard has yet to be determined. Oberdörster et al. (2006) suggested that toxicity studies should not simply focus on human and wildlife but should also examine benthic and soil flora and fauna, because they make up the basis of food chains. Biological systems did not evolve alongside the nanoparticles that are now being manufactured and released (Moore 2006). Different reactions to nZVI may be found in some lower organisms.

The Royal Commission on Environmental Pollution (2008) summed up the current approach to potential implications from nanomaterials:

While there have been no significant events that would lead us to suppose that the contemporary introduction of novel materials is a source of environmental hazard, we are acutely aware of past instances where new chemicals and products, originally thought to be entirely benign, turned out to have very high environmental and public health costs.

## Societal Issues

Most societal issues are based on the unknown risks of using nanoscale materials for site remediation. At one end of the spectrum, some nongovernmental groups invoked the precautionary principle in an attempt to halt all use of the technology until proven safe. In early 2003, the ETC Group called for the precautionary principle to be applied to nanotechnology (ETC Group 2003). They based their concerns on Eric Drexler’s concept of multiple nano-scale machines that might self-replicate and change matter into “gray goo” (Drexler 1986). Drexler later clarified this image (Phoenix and Drexler 2004), but not before Prince Charles of England became concerned enough about the risks of nanotechnology to ask the Royal Society to examine the implications of nanotechnology. In one part of their report (Royal Society and Royal Academy of Engineering 2004), the Royal Society came out strongly against the use of nanomaterials for remediation.

We recommend that the use of free (that is, not fixed in the matrix) manufactured nanoparticles and environmental applications such as remediation be prohibited until appropriate research has been undertaken and it can be demonstrated that the potential benefits outweigh the potential risks.

In contrast, the European Commission’s Scientific Committee on Emerging and Newly Identified Health Risks in 2005 (European Commission 2005) listed environmental remediation technology as one of nanotechnology’s benefits. This group also called for risk-related research.

In a position paper, the Québec Commission (Commission de l’Ethique de la Science et la Technologie 2006) indicated that

[T]he biggest source of potential environmental exposure is the use of nanoparticles in sanitizing contaminated groundwater and soil; concerns have been raised about the impact the high reactivity of nanoparticles might have on plants, animals, micro-organisms, and ecosystems.

The report noted “the importance of increasing the amount of research on the potential environmental consequences of nanotechnology in order to determine which substances may be hazardous.” Other risk framework documents have recommended research into the toxicity, fate and transport, and bioaccumulation of released nanomaterials (Maynard and Aitken 2006). A U.S. EPA white paper (U.S. EPA 2007) pointed out the positive aspects of using nanomaterials in environmental remediation while also calling for research on the possible negative effects.

In June 2007, DuPont and Environmental Defense released their nano risk framework (Medley and Walsh 2007). They chose ZVI nanoparticles as a case study. After going through the steps in the framework to assess the potential risk of using this technology, DuPont (2007) decided it “would not consider using this technology at a DuPont site until the end products of the reactions following injection, or following a spill, are determined and adequately assessed.” DuPont did not use their full output worksheet in this case study because of the lack of environmental, health, and safety data.

Although there is no consensus among these various reports on nanotechnology risk management, no doubt is expressed about the potential efficacy of the technology. However, the concerns over safety may limit the widespread deployment of nanoremediation. The reports cited above, as well as other published reports, consistently call for research specific to the possible risks of using nanotechnology in environmental remediation applications. The consensus is caution, not precaution, and, in the absence of definitive risk data, the technology is generally viewed as more beneficial than harmful.

## Recommendations

### Develop analytical tools to measure and monitor manufactured nanoparticles in the environment

Currently, standard methods to readily detect and monitor nano-particles in the environment do not exist. There are only a few quantitative analytical techniques for measuring nanoparticles in environmental systems, and most of these are time-consuming and require expensive equipment and expertise. Because there is no regulatory requirement to monitor environmental nanoparticles, or other particles such as those in drinking water, there is a critical lack of data and information about the occurrence and fate of nanoparticles once they are released into the environment. Some models and extrapolations attempt to quantify the amount of nanoparticles in various environmental systems. However, these models are based on estimates of nanoparticles released into the environment and have not been calibrated with actual measurements in the field (Mueller and Nowack 2008). It is difficult, if not impossible, to extrapolate the toxicity and pathology of nanoparticles at the ecosystem level until sufficient baseline data on these particles are gathered (Moore 2006). There are also no biomarkers that can be used to track nanoparticles as part of a biological monitoring program; although existing regulatory toxicity tests could be appropriate for nanoparticles, a risk analysis would not be possible without proper measurement of the concentrations of nanoparticles in the environment (Handy et al. 2008).

### Increase research to evaluate the effects of nanoparticles on the full ecosystem

Nowack and Bucheli (2007) concluded that results from ecotoxicologic studies show that organisms are affected by certain nanoparticles under certain environmental conditions. However, the studies were conducted using elevated concentrations of pristine nanoparticles. The authors recommended that future studies estimate the exposure to functionalized nanoparticles, because most manufactured nanoparticles are functionalized, which changes their behavior. Changes by environmental factors such as light, oxidants, and microorganisms—which result in chemical or biological modifications or degradation of the functionalized surface or coating of the surface with natural compounds—are important processes that have not been studied thoroughly (Nowack and Bucheli 2007). In addition, most nanoparticles are released embedded in a matrix and not as single nanoparticles (Koehler et al. 2007). It is important to study nanoparticles in the form in which organisms in the ecosystem and humans might be exposed to them.

The properties that can be harmful to the environment are the very same properties that are advantageous and exploited during treatment and remediation regimes. For instance, the catalytic properties of nanoparticles that induce the degradation of pollutants can also induce a toxic response when taken up by cells. In addition, the high sorption capacity of nanoparticles that is used to remove organic and inorganic pollutants from groundwater may also sequester and transport other pollutants in the environment (Nowack 2008). As such, more work is needed on transfers in environmental systems, for example, from the environment to the organism and throughout the trophic structure.

Further research is needed to develop and understand the mechanisms affecting the fate and transport of manufactured nanoparticles in water, soil, and sediments; their interactions with each other, other manufactured nanoparticles, suspended solids, and dissolved organic material; and how these interactions are influenced by different environmental variables. The potential for manufactured nanoparticles to act as carriers for other environmental contaminants also requires further examination.

### Improve engineering applications using nanotechnology for *in situ* remediation

There is a need to develop “smarter” nanomaterials for remediation. For example, new coatings or functional groups could enhance mobility in groundwater. More sophisticated nano-materials may have the ability to perform several functions, such as catalyzing several different pollutant reactions on the same particle or interacting with both hydrophobic and hydrophilic pollutants. We can build in self-termination for active nanoparticles so they become benign after their remediation function is finished; design nanoparticles that destroy a wide spectrum of pollutants; and improve delivery systems for injecting nanoparticles into contaminated groundwater plumes.

All these engineering improvements can increase the ability of this technology to remediate more of the world’s hazardous waste sites. Engineering more effective particles can improve the ability to reach and remediate pollutant plumes and minimize potential harm.

## Conclusions

*In situ* nanoremediation methods entail the application of reactive nanomaterials for transformation and detoxification of pollutants *in situ*. These nanomaterials have properties that enable both chemical reduction and catalysis to mitigate the pollutants of concern. No groundwater is pumped out for above-ground treatment, and no soil is transported to other places for treatment and disposal. Nanoscale Fe particles are effective for the remediation and transformation of a variety of environmental contaminants. Because of the high cost and lengthy operating periods for pump-and-treat remedies, *in situ* ground-water treatment technologies are increasing. The number of actual applications of nZVI is increasing rapidly. Only a fraction of the projects have been reported, and new projects show up regularly. Although the technology is likely a beneficial replacement of current practices for site remediation, potential risks are poorly understood. The factors and processes affecting ecotoxicity are complex, and knowledge of the potential impacts of manufactured nanoparticles in the environment on human health is still limited. Most societal issues are based on these unknown risks of using nanoscale materials for site remediation.

Nanoremediation has the potential to reduce the overall costs of cleaning up large-scale contaminated sites, reduce cleanup time, eliminate the need for treatment and disposal of contaminated dredged soil, and reduce some contaminant concentrations to near zero, and it can be done *in situ*. In order to prevent any potential adverse environmental impacts, proper evaluation, including full-scale ecosystem-wide studies, of these nanoparticles needs to be addressed before this technique is used on a mass scale.

## Figures and Tables

**Table 1:** Percentage of total sites being remediated with respect to each media and contaminant group (U.S. EPA 2004).

	Media			Contaminant group		
Type of site	Groundwater	Soil	Sediment	VOCs	Metals	SVOCs
NPL	83	78	32	78	77	71
RCRA-CA	82	61	6	67	46	32
DOD	63	77	18	64	72	57
DOE	72	72	72	38	55	38

SVOCs, semivolatile VOCs.

## References

[b1-ehp-117-1823] Adams LK, Lyon DY, Alvarez PJJ (2006). Comparative ecotoxicity of nanoscale TiO_2_, SiO_2_ and ZnO water suspensions. Water Res.

[b2-ehp-117-1823] American Recovery and Reinvestment Act (2009). http://frwebgate.access.gpo.gov/cgi-bin/getdoc.cgi?dbname=111_cong_bills&docid=f:h1enr.pdf.

[b3-ehp-117-1823] Auffan M, Decome L, Rose J, Orsiere T, De Meo M, Briois V (2006). In vitro interactions between DMSA-coated maghemite nanoparticles and human fibroblast: a physicochemical and cytogenotoxical study. Environ Sci Technol.

[b4-ehp-117-1823] Biswas P, Wu C-Y (2005). Nanoparticles and the environment. J Air Waste Manag.

[b5-ehp-117-1823] Boxall ABA, Tiede K, Chaudhry Q (2007). Engineered nanomaterials in soils and water: how do they behave and could they pose a risk to human health?. Nanomedicine.

[b6-ehp-117-1823] Cao J, Elliott D, Zhang W-X (2005). Perchlorate reduction by nanoscale iron particles. J Nanopart Res.

[b7-ehp-117-1823] CERCLA (1980). Title 42 – The Public Health and Welfare, Chapter 103- Comprehensive Environmental Response, Compensation, and Liability. http://frwebgate.access.gpo.gov/cgi-bin/usc.cgi?ACTION=BROWSE&TITLE=42USCC103.

[b8-ehp-117-1823] Commission de l’Ethique de la Science et la Technologie (2006). Ethics and Nanotechnology: A Basis for Action.

[b9-ehp-117-1823] Continental Remediation LLC (2009). Home Heating Oil Remediation—New Jersey.

[b10-ehp-117-1823] Dhawan A, Taurozzi JS, Pandey AK (2006). Stable colloidal dispersion of C60 fullerenes in water: evidence for genotoxicity. Environ Sci Technol.

[b11-ehp-117-1823] Drexler E (1986). Engines of Creation: The Coming Era of Nanotechnology.

[b12-ehp-117-1823] DuPont (2007). Nanomaterial Risk Assessment Worksheet.

[b13-ehp-117-1823] Elliott DW, Zhang WX (2001). Field assessment of nanoscale bimetallic particles for groundwater treatment. Environ Sci Technol.

[b14-ehp-117-1823] ETC Group (2003). The Big Down: From Genomes to Atoms.

[b15-ehp-117-1823] European Commission (2005). Scientific Committee on Emerging and Newly Identified Health Risks (SCENIHR) Opinion on the Appropriateness of Existing Methodologies to Assess the Potential Risks Associated with Engineered and Adventitious Products of Nanotechnologies.

[b16-ehp-117-1823] Fortner JD, Lyon DY, Sayes CM, Boyd AM, Falkner JC, Hotze EM (2005). C60 in water: nanocrystal formation and microbial response. Environ Sci Technol.

[b17-ehp-117-1823] Gavaskar A, Tatar L, Condit W (2005). Cost and Performance Report Nanoscale Zero-Valent Iron Technologies for Source Remediation.

[b18-ehp-117-1823] Gilbert B, Lu G, Kim CS (2007). Stable cluster formation in aqueous suspensions of iron oxyhydroxide nanoparticles. J Colloid Interface Sci.

[b19-ehp-117-1823] Glazier R, Venkatakrishnan R, Gheorghiu F, Walata L, Nash R, Zhang W (2003). Nanotechnology takes root. Civil Eng.

[b20-ehp-117-1823] Handy RD, von der Kammer F, Lead JR, Hassellöv M, Owen R, Crane M (2008). The ecotoxicology and chemistry of manufactured nanoparticles. Ecotoxicology.

[b21-ehp-117-1823] Henn KW, Waddill DW (2006). Utilization of nanoscale zerovalent iron for source remediation—a case study. Remediation.

[b22-ehp-117-1823] Hochella MF, Moore JN, Putnis CV, Putnis A, Kasama T, Eberl DD (2005). Direct observation of heavy metal-mineral association from the Clark Fork River Superfund Complex: implications for metal transport and bioavailability. Geochim Cosmochim Acta.

[b23-ehp-117-1823] Kanel SR, Greeneche JM, Choi H (2006). Arsenic (V) removal from groundwater using nanoscale zerovalent iron as a colloidal reactive barrier material. Environ Sci Technol.

[b24-ehp-117-1823] Keenan CR, Sedlak DL (2008). Factors affecting the yield of oxidants from the reaction of nanoparticulate zerovalent iron and oxygen. Environ Sci Technol.

[b25-ehp-117-1823] Kersting AB, Efurd DW, Finnegan DL, Rokop DJ, Smith DK, Thompson JL (1999). Migration of plutonium in ground water at the Nevada Test Site [Letter]. Nature.

[b26-ehp-117-1823] Koehler A, Som C, Helland A, Gottschalk F (2007). Studying the potential release of carbon nanotubes throughout the application life cycle. J Cleaner Prod.

[b27-ehp-117-1823] Labrenz M, Drusche GK, Thomsen-Ebert T, Gilbert B, Welch SA, Kemner KM (2000). Formation of sphalerite (ZnS) deposits in natural biofilms of sulfate-reducing bacteria. Science.

[b28-ehp-117-1823] Lovern SB, Strickler JR, Klaper R (2007). Behavioral and physiological changes in *Daphnia magna* when exposed to nanoparticle suspensions (titanium dioxide, nano-C_60_ and C_60_HxC_70_Hx). Environ Sci Technol.

[b29-ehp-117-1823] Macé C, Desrocher S, Gheorghiu F, Kane A, Pupeza M, Cernik M (2006). Nanotechnology and groundwater remediation: a step forward in technology understanding. Remediation.

[b30-ehp-117-1823] Mackay CE, Johns M, Salatas JH, Bessinger B, Perri M (2006). Stochastic probability modeling to predict the environmental stability of nanoparticles in aqueous suspension. Integr Environ Assess Manag.

[b31-ehp-117-1823] Madden AS, Hochella MF, Luxton TP (2006). Insights for size-dependent reactivity of hematite nanomineral surfaces through Cu^2+^ sorption. Geochim Cosmochim Acta.

[b32-ehp-117-1823] Matheson LJ, Tratnyek PG (1994). Reductive dehalogenation of chlorinated methanes by iron metal. Environ Sci Technol.

[b33-ehp-117-1823] Maynard AD, Aitken RJ, Butz T, Colvin V, Donaldson K, Oberdörster G (2006). Safe handling of nanotechnology. Nature.

[b34-ehp-117-1823] Medley T, Walsh S (2007). Nano Risk Framework: Environmental Defense–DuPont Partnership.

[b35-ehp-117-1823] Moore MN (2006). Do nanoparticles present ecotoxicological risks for the health of the aquatic environment?. Environ Sci Technol.

[b36-ehp-117-1823] Mueller NC, Nowack B (2008). Exposure modeling of engineered nanoparticles in the environment. Environ Sci Technol.

[b37-ehp-117-1823] National Nanotechnology Initiative (2009). What Is Nanotechnology?.

[b38-ehp-117-1823] Nel A, Xia T, Mädler L, Li N (2006). Toxic potential of materials at the nanolevel. Science.

[b39-ehp-117-1823] Novikov AP, Kalmykov SN, Utsunomiya S, Ewing RC, Horreard F, Merkulov A (2006). Colloid transport of plutonium in the far-field of the Mayak Production Association, Russia. Science.

[b40-ehp-117-1823] Nowack B, Krug H (2008). Pollution prevention and treatment using nanotechnology. Nanotechnology, Vol 2 Environmental Aspects.

[b41-ehp-117-1823] Nowack B, Bucheli TD (2007). Occurrence, behavior and effects of nanoparticles in the environment. Environ Pollut.

[b42-ehp-117-1823] Oberdörster E, Zhu S, Blickley TM, McClellan Green P, Haasch ML (2006). Ecotoxicology of carbon-based engineered nanoparticles: effects of fullerene (C_60_) on aquatic organisms. Carbon.

[b43-ehp-117-1823] O’Hara S, Krug T, Quinn J, Clausen C, Geiger C (2006). Field and laboratory evaluation of the treatment of DNAPL source zones using emulsified zerovalent iron. Remediation.

[b44-ehp-117-1823] Otto M, Floyd M, Bajpai S (2008). Nanotechnology for site remediation. Remediation.

[b45-ehp-117-1823] PARS Environmental Inc (2004). NanoFe™: An Innovative Remediation Technology for Soils and Groundwater.

[b46-ehp-117-1823] Phenrat T, Saleh N, Sirk K, Tilton RD, Lowry GV (2007). Aggregation and sedimentation of aqueous nanoscale zerovalent iron dispersions. Environ Sci Technol.

[b47-ehp-117-1823] Phoenix C, Drexler E (2004). Safe exponential manufacturing. Nanotechnology.

[b48-ehp-117-1823] Project on Emerging Nanotechnologies (2009). Nanoremediation Map.

[b49-ehp-117-1823] RCRA (Resource, Conservation, and Recovery Act) (2002). http://epw.senate.gov/rcra.pdf.

[b50-ehp-117-1823] Rickerby DG, Morrison M (2007). Nanotechnology and the environment: a European perspective. Sci Technol Adv Mater.

[b51-ehp-117-1823] Royal Commission on Environmental Pollution (2008). Novel Materials in the Environment: The Case of Nanotechnology.

[b52-ehp-117-1823] Royal Society and Royal Academy of Engineering (2004). Nanoscience and Nanotechnologies: Opportunities and Uncertainties.

[b53-ehp-117-1823] Saleh N, Sirk K, Liu YQ, Phenrat T, Dufour B, Matyjaszewski K (2007). Surface modifications enhance nanoiron transport and NAPL targeting in saturated porous media. Environ Eng Sci.

[b54-ehp-117-1823] Small Business Liability Relief and Brownfields Revitalization Act (2002). http://frwebgate.access.gpo.gov/cgi-bin/getdoc.cgi?dbname=107_cong_public_laws&docid=f:publ118.107.

[b55-ehp-117-1823] Sposito G (1989). The Chemistry of Soils.

[b56-ehp-117-1823] Sun YP, Li X, Cao J, Zhang W, Wang HP (2006). Characterization of zerovalent iron particles. Adv Colloid Interface Sci.

[b57-ehp-117-1823] Theron J, Walker JA, Cloete TE (2008). Nanotechnology and water treatment: applications and emerging opportunities. Crit Rev Microbiol.

[b58-ehp-117-1823] Tratnyek PG, Johnson RL (2006). Nanotechnologies for environmental cleanup. Nanotoday.

[b59-ehp-117-1823] Tratnyek PG, Scherer MM, Johnson TL, Matheson LJ, Tarr MA (2003). Permeable reactive barriers of iron and other zerovalent metals. Chemical Degradation Methods for Wastes and Pollutants, Environmental and Industrial Applications.

[b60-ehp-117-1823] U.S. EPA (2001). Cost Analysis for Selected Groundwater Cleanup Projects: Pump and Treat Systems and Permeable Reactive Barriers.

[b61-ehp-117-1823] U.S. EPA (2004). Cleaning Up the Nation’s Waste Sites: Markets and Technology Trends.

[b62-ehp-117-1823] U.S. EPA (2007). Nanotechnology White Paper.

[b63-ehp-117-1823] U.S. EPA (2008a). FY 2007 Superfund Annual Report. Building on Success: Protecting Human Health and the Environment.

[b64-ehp-117-1823] U.S. EPA (2008b). Nanotechnology for Site Remediation: Fact Sheet.

[b65-ehp-117-1823] U.S. EPA (Environmental Protection Agency) (2009). Superfund Sites.

[b66-ehp-117-1823] Valko M, Morris H, Cronin MTD (2005). Metals, toxicity, and oxidative stress. Curr Med Chem.

[b67-ehp-117-1823] Van der Zee C, Roberts DR, Rancourt DG, Slomp CP (2003). Nanogoethite is the dominant reactive oxyhydroxide phase in lake and marine sediments. Geology.

[b68-ehp-117-1823] Vilks P, Frost LH, Bachinski DB (1997). Field-scale colloid migration experiments in a granite fracture. J Contam Hydrol.

[b69-ehp-117-1823] Villalobos M, Toner B, Bargar J, Sposito G (2003). Characterization of the manganese oxide produced by *Pseudomonas putida* strain MnB. Geochim Cosmochim Acta.

[b70-ehp-117-1823] Waite TD, Schafer AI, Fane AG, Heuer A (1999). Colloidal fouling of ultrafiltration membranes: impact of aggregate structure and size. J Colloid Interface Sci.

[b71-ehp-117-1823] Wang CB, Zhang WX (1997). Synthesizing nanoscale iron particles for rapid and complete dechlorination of TCE and PCBs. Environ Sci Technol.

[b72-ehp-117-1823] Watlington K (2005). Emerging Nanotechnologies for Site Remediation and Wastewater Treatment.

[b73-ehp-117-1823] Wiesner MR, Lowry GV, Alvarez P, Dionysios D, Biswas P (2006). Assessing the risks of manufactured nanomaterials. Environ Sci Technol.

[b74-ehp-117-1823] Zhang W-X (2003). Nanoscale iron particles for environmental remediation: an overview. J Nanopart Res.

[b75-ehp-117-1823] Zhang WX (2005). Nanotechnology for Water Purification and Waste Treatment. Frontiers in Nanotechnology.

[b76-ehp-117-1823] Zhang W-X, Elliott DW (2006). Applications of iron nanoparticles for groundwater remediation. Remediation.

